# Efficacy of two once-daily methylphenidate formulations compared across dose levels at different times of the day: Preliminary indications from a secondary analysis of the COMACS study data

**DOI:** 10.1186/1471-244X-4-28

**Published:** 2004-09-30

**Authors:** Edmund JS Sonuga-Barke, James M Swanson, David Coghill, Heleen H DeCory, Simon J Hatch

**Affiliations:** 1 The Developmental Brain-Behaviour Unit, University of Southampton, Southampton, SO17 1BJ. UK; 2 Child Development Center, University of California at Irvine, Irvine, CA 19722. USA; 3 The Department of Psychiatry, The University of Dundee, Dundee, DD1 9SY. UK; 4 Medical and Regulatory Affairs, Celltech Americas, Inc., Rochester, NY 14603. USA

## Abstract

**Background:**

Methylphenidate (MPH) is commonly prescribed in the treatment of Attention-Deficit/Hyperactivity Disorder or ADHD. Concerta and Metadate CD are once-daily formulations of MPH using different delivery mechanisms resulting in different pharmacokinetic profiles. A recent study (COMACS) showed that for near-milligram (mg) equivalent daily doses, Metadate CD provides greater symptom control in the morning (1.5 through 4.5 hours post-dose), while Concerta provides greater control in the early evening (12 hours post-dose). Non-inferential comparison of effects for different dose levels of the two formulations suggested that equivalent levels of morning symptom control could be obtained with lower daily doses of Metadate CD than Concerta; the situation being reversed in the evening. The current paper presents a secondary analysis that provides a statistical test of these observations.

**Method:**

The COMACS study was a multi-center, double-blind crossover study of Metadate CD, Concerta and placebo with each treatment administered for 1 week. Children were assigned on the basis of their pre-trial dosage to either high (Metadate CD 60 mg; Concerta 54 mg), medium (Metadate CD 40 mg; Concerta 36 mg) or low doses (Metadate CD 20 mg; Concerta 18 mg) of MPH, and attended a laboratory school on the 7th day for assessment at 7 sessions across the day. For the *post-hoc *comparisons across dose levels presented here, total SKAMP scores with the active treatments (adjusted for placebo response) were analyzed using an analysis of covariance, with a combined measure modeling placebo response across all time period as the covariate.

**Results:**

Symptom control from 1.5 through 6.0 hours post-dose was as good with lower doses of Metadate CD (20 and 40 mg) as with higher doses of Concerta (36 and 54 mg, respectively). Lower daily doses of Concerta (18 and 36 mg) and higher doses of Metadate CD (40 and 60 mg, respectively) gave equivalent control at 7.5 and 12 hours with Metadate CD giving better control from1.5 through 6.0 hours post-dose.

**Conclusions:**

Different delivery profiles of Metadate CD and Concerta can be exploited to limit total daily exposure to MPH while at the same targeting a specific, especially clinically significant, period of the day. These results need to be confirmed in a study in which children are randomly allocated to different dose levels of the two formulations and plasma MPH concentrations are assessed simultaneously.

## Background

Attention Deficit /Hyperactivity Disorder (ADHD) is a relatively common early onset developmental condition characterised by a pervasive and persistent pattern of age inappropriate and debilitating inattention, impulsiveness and overactivity. It is reported to affect between 3 and 6 percent of the childhood population and, if untreated, to be associated with a poor prognosis in adolescence and adulthood [1,2]. Methylphenidate (MPH) remains a pharmacological treatment of first choice for children with ADHD [3]. Historically, effective 'all-day' management of symptoms has relied on the use of multiple doses (typically two or three) of immediate release (IR) MPH spread out across the day (early morning, midday and evening)[4]. The use of IR formulations in this way combines all-day coverage with the opportunity to tailor doses at different times of the day to meet the specific needs of children. However, there is evidence that multiple dosing leads to problems with adherence especially during the school day where children receiving medication may feel stigmatised by their classmates [5]. Once-a-day sustained release (SR) formulations have been licensed in the US for some time but the early formulations were not widely used because of the perceived lack of efficacy especially with regard to speed of onset [6]. In the last few years a second generation of more effective formulations (referred to here as extended release formulations – ER) have been licensed. These exploit a range of different delivery technologies and offer smooth patterns of symptom control across the day [7-9]. These new formulations represent a major advance in the clinical management of the condition and are popular with both patients and clinicians. Various ER formulations have been designed each with a different pharmacokinetic (PK) and pharmacodynamic (PD) profile that results in differing patterns of duration and timing of effect. Thus they have the potential to provide clinicians with the opportunity to simplify the dosing regime without loosing the ability to tailor treatment to the clinical profile of an individual patient. In order to exploit this opportunity clinicians need to be able to make informed decisions about the comparative benefits of differing doses of different formulations with different PK/PD profiles. Unfortunately, to date, there have been few head-to-head trials of these new ER formulations that provide the information required for this.

We recently reported the results of a randomised, placebo-controlled, head-to-head comparison of the pharmacodynamic (PD) properties of near mg-equivalent daily doses of two safe and effective [10,11] ER formulations of MPH in children (the COMACS study; [12]). Concerta (CON) was designed to replace three-times-a-day (TID) IR MPH and provide twelve-hour symptom coverage. It consists of an insoluble OROS™ tablet with an IR drug over coat. Twenty two percent of the dose is in the IR overcoat and 78% in the ER core, which is released, by an osmotic pump process [7,10]. Metadate CD (MCD) has a profile more in keeping with a two-times-a-day (BID) regime of IR MPH. It consists of a soluble capsule containing a mixture of IR MPH beads (30% of the total dose) and ER MPH beads coated with a controlled-release polymer to deliver MPH gradually over the extended period (70% of the total dose) [8,11]. Low (20 mg MCD and 18 mg CON), medium (40 mg MCD and 36 mg CON), and high (60 mg MCD and 54 mg CON) doses are available for each formulation and have been demonstrated to deliver equivalent levels of total exposure to MPH in adults [13] (as shown by comparable AUCs and C_max_). Recently a 10 and 30 mg dose of MCD and a 27 mg dose of CON were licensed, but these were not available during the COMACS study. As a result of the design differences, MCD releases 50% more IR MPH in the initial bolus delivery process than CON for a near mg-equivalent total daily doses. Furthermore although the amount of ER MPH is the same for near mg-equivalent doses the pattern of delivery differs with MPH release front-loaded with MCD (starting at 1 hour) and more back-loaded with CON.

The different IR/ER ratios and time-course for dissolution of the two extended-release portions of the formulations result in distinctly different plasma concentration vs. time profiles in adults when compared at near-mg equivalent total doses [13]. Concentrations of MPH are significantly higher for MCD than for CON for up to 6 hours after dosing, and, by contrast, concentrations of MPH are significantly higher for CON from 8 through 12 hrs after dosing. Differences in plasma concentration vs. time profiles are expected to occur in children as the PK of MPH in adults and children are qualitatively similar (i.e. there are no reported age-related differences in absorption, distribution, metabolism and excretion of MPH between these populations [14-16]). In agreement, the plasma concentration vs. time profile for MCD in children is consistent with that observed in adults [8]. Results of the COMACS study demonstrated that the PD patterns of the two formulations in children mirrored their expected differences in plasma concentration vs. time profiles. This meant that at each of the three near-mg equivalent daily dose levels, MCD produced a greater reduction in symptoms during the morning (up to six hours from drug administration) while CON produced superior control in the early evening (i.e., at 12 hours post-dose). However, the two formulations were equivalent in their effects during the afternoon between 6 and 7.5 hours post dose [12].

The publication of the main analysis of the COMACS study data has led to a number of suggestions for alternative analyses that would usefully address questions of the clinical utility of the two formulations. Although from a scientific point of view the aim of the COMACS study – to compare across-the-day PD profiles of the two formulations – was best served by a comparison of bio-equivalent (i.e. AUC-equivalent) total daily doses, other strategies for selecting comparator doses could have been legitimately followed. For instance, it has been suggested that comparators could have been matched on the basis of the size of the IR component dose rather than the total daily dose (see Table 1 for IR and ER components of the MCD and CON). In this regard it is interesting that an informal comparison of the relative efficacy of MCD and CON at each dose level suggested that equivalent morning symptom control would possibly be obtained at doses with a near mg-equivalent IR component but with a lower total daily dose of MCD than CON, while the situation was reversed in the evening (that is, lower daily doses of CON could have equivalent efficacy to higher doses of MCD). In each case, the COMACS study data suggested that this targeted control would be gained at the cost of efficacy at other point(s) during the day. These across-the-day-changes in patterns of the relative efficacy of the two formulations are consistent with what would be expected on the basis of their IR/ER ratios, their drug delivery mechanisms, and their resulting predicted plasma concentration vs. time profiles.

**Table 1:** Amounts of Immediate-Release (IR) and Extended-Release (ER) Methylphenidate in Available Dosage forms of Metadate CD and Concerta^a^

	IR MPH	ER MPH
18 mg CON	4 mg	14 mg
20 mg MCD	6 mg	14 mg
36 mg CON	8 mg	28 mg
40 mg MCD (2 × 20 mg capsules)	12 mg	28 mg
54 mg CON	12 mg	42 mg
60 mg MCD (3 × 20 mg capsules)	18 mg	42 mg

^a^Recently, a 10- and 30-mg dose strength of Metadate CD and a 27-mg dose strength of Concerta have become available. These were not available at the time of the COMACS study.

These observations could be clinically important because it is expected that patients and their families may be willing to trade some degree of symptom control at certain times of the day if that allows them to reduce the overall dose of MPH. In such cases, clinicians will seek a dosing profile that allows them to target a specific period of the day that is especially important for a particular patient. By doing this they can retain the effectiveness of a higher dose component during these selected periods of the day while limiting total daily MPH exposure. For instance, in some cases clinicians, patients and parents may seek symptom control in the evening while being less concerned about the daytime. In other cases they may wish to focus on morning and afternoon symptom control.

Because the main aim of the COMACS study design was to compare the PD profiles of MCD and CON across the day, initial analyses were limited to within-subject comparisons between equivalent total daily doses (low, medium and high) of the formulations. Thus this primary analysis of the data did not include a cross-dose analysis, which would allow the relative efficacy of different daily doses of MCD and CON to be directly tested at different times of the day. The present paper presents a statistical cross-dose analysis of the efficacy of MCD and CON at different times of the day in order to test these observations directly.

The ideal way to test across-dose comparisons of different medications is to randomise patients to different dosing strata. In the COMACS study children were assigned to dosing strata on the basis of pre-trial dose levels. However, because the COMACS study was designed to have similar and substantial numbers of children in each dosing strata the data set still offers the opportunity for a preliminary *post-hoc *exploration of the PD profiles of different doses of MCD and CON. Furthermore, the inclusion of the placebo arm in the COMACS design enabled us to directly address one of the possible major confounds associated with across dose comparisons in this sort of stratified design. An inspection of the published COMACS data revealed that the placebo scores measured at different sessions across the day were higher in the high dose group, than the medium or low dose groups. This suggests that children on higher doses may have had a more severe expression of the disorder making cross-group comparisons complicated. In the analysis reported in the current paper these placebo scores were used to adjust the treatment outcome scores to take account of this.

A number of specific across-dose comparisons were made. First, the lower daily doses (20 mg and 40 mg) of MCD were compared to the higher daily doses of CON (36 mg and 54 mg respectively). The comparison between MCD 20 mg and CON 36 mg is of particular interest to clinicians as these are the suggested dose substitutions for IR MPH 10 mg BID and 10 mg TID, respectively. On the basis of the initial observations of the COMACS study it was predicted that MCD 20 and CON 36 mg, and MCD 40 and CON 54 mg would produce equivalent control in the morning (from 1.5 through 4.5 hours post-dose). In contrast, at these dose levels it was predicted that CON would produce significantly greater effect as the day wore on (from 6 hours onwards). Second, lower daily doses (18 mg and 36 mg) of CON were compared with higher doses of MCD (40 mg and 60 mg respectively). We tested the prediction that at these dosing levels MCD would demonstrate greater efficacy only in the morning and early afternoon (from 1.5 through 6 hours); so that despite the lower total daily dosing levels, CON, designed to be effective over a 12 hour period, would still be more effective than MCD, designed to be effective over an 8 hour period, at reducing symptoms in the early evening (i.e., at 12 hours).

## Methods

### Clinical materials

Metadate^® ^CD (methylphenidate HCl, USP) Extended-Release Capsules (MCD) were obtained from Eurand Americas, Inc (Vandalia, OH), while Concerta^® ^(methylphenidate HCl) Extended-release Tablets (CON) were obtained from Alza Corporation (Mountain View, CA). For a detailed description of the preparation of clinical materials see Swanson et al., 2004 [12].

### Patients

Six to 12 year old children, with interview-confirmed-diagnoses of ADHD who were being treated with MPH in doses of between 10 to 60 mg/day (5 mg to 20 mg per administration, one to three times a day) were recruited for the trial. Children were deemed otherwise healthy on the basis of an extensive medical history and physical examination. Children were excluded if they had an IQ below 80 or the inability to follow or understand study instructions. Other standard exclusion criteria for MPH drug trials applied [12]. Children provided signed assent, and their legal guardians signed an IRB-approved consent form. A total of 214 patients were screened for participation into the study and 184 patients (74 percent of which were male) were stratified across the three dose levels based on their previously established clinical doses of MPH. Eighty-two percent of the patients met the criteria for ADHD-Combined Type with a further 15 percent meeting the criteria for Inattentive Type. Approximately 25% of children had a co-morbid condition (e.g., anxiety and oppositional defiant disorder). At prescreening, approximately 91% of the patients were on once-a-day dosing regimens. Of the remainder 7.6% were taking BID and 1.6% TID IR MPH. Of the 184 subjects entering the study, 157 received all three levels of treatment and participated in all seven classroom sessions. The demographic characteristics of the sample of patients that completed all three treatments (n = 157) were not different than those reported for the full sample.

### Design

This was a ten-site, double-blind, placebo-controlled crossover study comparing three treatment conditions: MCD, CON, and placebo (PLA). The study was conducted in accordance with the principle of the Declaration of Helsinki and its amendments and the International Committee on Harmonization Guidelines on Good Clinical Practice. Dose-level assignment was made according to pre-existing daily dosing requirement for MPH and children remained at the dose-level assigned for the entire study duration. Children treated with low doses (≤ 15 mg/day IR or 20 mg/day ER) of MPH were randomized to receive a daily dose of MCD 20 mg, CON 18 mg or PLA; those treated with medium doses (>10 to ≤ 30 mg/day IR or >20 to ≤ 40 mg/day ER) of MPH were randomized to receive MCD 40 mg, CON 36 mg or PLA; and children treated with high doses (> 30 mg/day IR or >40 mg/day ER) of MPH were randomized to receive MCD 60 mg, CON 54 mg, or PLA. Each of the three treatments was administered for 7 days (in randomized order) without an intervening washout period. Assessments took place in the laboratory school on Days 7, 14, and 21 (for a detailed description of the laboratory classroom day see Swanson et al., 2004 [12]). Two trained observers assessed patients during each classroom session on the Swanson, Kotkin, Atkins, M/Flynn, Pelham Scale (SKAMP; [17,18]) on the basis of a 1.5-hour cycle of activities with separate assessments of Attention and Deportment being made at 0, 1.5, 3.0, 4.5, 6.0, 7.5 and then 12 hours after drug administration. Because Attention and Deportment scores were highly correlated in the original COMACS analysis, these subscales were combined in the current analysis for ease of presentation.

### Statistical analyses

A preliminary factorial Analysis of Variance (ANOVA) using the SPSS General Linear Model was conducted in order to confirm that the pattern of effects of Treatment (CON, MCD, PLA), Dose (low, med, high) and Session (0, 1.5, 3.0, 4.5, 6, 7.5 and 12 hours) found in the Swanson et al. (2004) paper [12] held when the individual SKAMP Attention and Deportment scales were combined to provide a composite score. In order to test the specific across-dose predictions Analysis of Covariance (ANCOVA) was used to make the four comparisons outlined above. In each case, Treatment/Dose (analysis 1-MCD 20 mg vs. CON 36 mg; analysis 2-MCD 40 mg vs. CON 54 mg; analysis 3-MCD 40 mg vs. CON 18 mg; analysis 4-MCD 60 mg vs. CON 36 mg) was the between-subjects independent factor. Session was the within- subject factor, and the dependent variable was total SKAMP score. Children's active drug SKAMP scores were adjusted to take account of their behaviour on PLA using a weighted combined SKAMP score for all observation points. Weighting was determined by principle components analysis and was similar for each observation point. Other between-subjects factors included in the initial COMACS study data analysis (including Site and Sequence of Drugs) were excluded from the current analysis. The GLM option that utilizes data from just those subjects with complete data (i.e., those cases without missing data) was selected for each separate analysis.

## Results

### Preliminary analysis

Table 2 shows the total SKAMP scores for each observation sessions at each dose level of CON, MCD and placebo. There were significant main effects of treatment, F (2, 306) = 92.06; p < 0.001, and session, F (6, 918) = 34.70; p < 0.001, and an interaction between treatment and session, F (12, 1836) = 45.21; p < 0.001. Planned comparisons demonstrated that the relative efficacy of the two formulations in relation to PLA was as described by Swanson et al. [12] for separate SKAMP deportment and attention scales: CON = MCD < PLA at the time of dose delivery, MCD > CON > PLA for 1.5, 3 and 4.5 hours, MCD = CON > PLA for session 6 and 7.5 hours and MCD = PLA < CON at 12 hours. For a discussion on the superiority of placebo immediately after dosing, the reader is referred to Swanson et al [12].

**Table 2:** Mean (± SD) SKAMP Total Scores at Each Observation Session for Each Treatment at Each Dose Level^a^

	MCD			CON			PLA		
Observation Session (hrs)	Low	Med	High	Low	Med	High	Low	Med	High
0	18.48 (11.82)	20.88 (12.95)	19.91 (13.15)	18.04 (10.13)	19.14 (12.14)	21.47 (13.06)	13.58 (9.72)	16.02 (11.84)	13.96 (11.14)
1.5	11.44 (7.99)	10.98 (8.62)	6.55 (5.85)	14.04 (9.85)	14.86 (12.01)	11.34 (9.71)	19.10 (12.83)	19.47 (12.56)	18.88 (13.48)
3.0	12.57 (9.92)	11.03 (9.66)	7.31 (6.10)	16.44 (12.43)	15.29 (12.72)	12.62 (11.00)	21.47 (14.61)	20.98 (14.11)	22.11 (14.10)
4.5	13.46 (11.53)	12.39 (10.32)	9.15 (8.62)	17.55 (13.37)	15.09 (12.60)	13.55 (11.91)	20.23 (11.92)	22.09 (15.46)	23.44 (12.55)
6.0	16.08 (13.27)	14.47 (11.53)	10.30 (9.71)	17.00 (12.12)	14.28 (11.73)	12.04 (11.62)	22.98 (12.79)	22.15 (13.91)	26.02 (14.56)
7.5	15.85 (11.21)	17.26 (13.63)	14.29 (12.55)	18.62 (12.66)	15.19 (13.47)	13.47 (12.97)	23.54 (12.96)	23.13 (14.72)	24.48 (14.68)
12.0	20.44 (13.75)	20.28 (15.02	19.85 (14.41)	16.90 (13.36)	17.81 (13.84)	16.74 (14.98)	19.45 (13.46)	20.73 (13.46)	22.02 (15.17)

^a^Lower SKAMP scores indicate greater efficacy

CON = Concerta; MCD = Metadate CD; PLA = placebo. Low = Low Dose (CON 18 mg; MCD 20 mg), Med = Medium Dose (CON 36 mg; MCD 40 mg), Hi = High Dose (CON 54 mg ; MCD 60 mg).

### Comparison across dose levels

**Figure 1:**
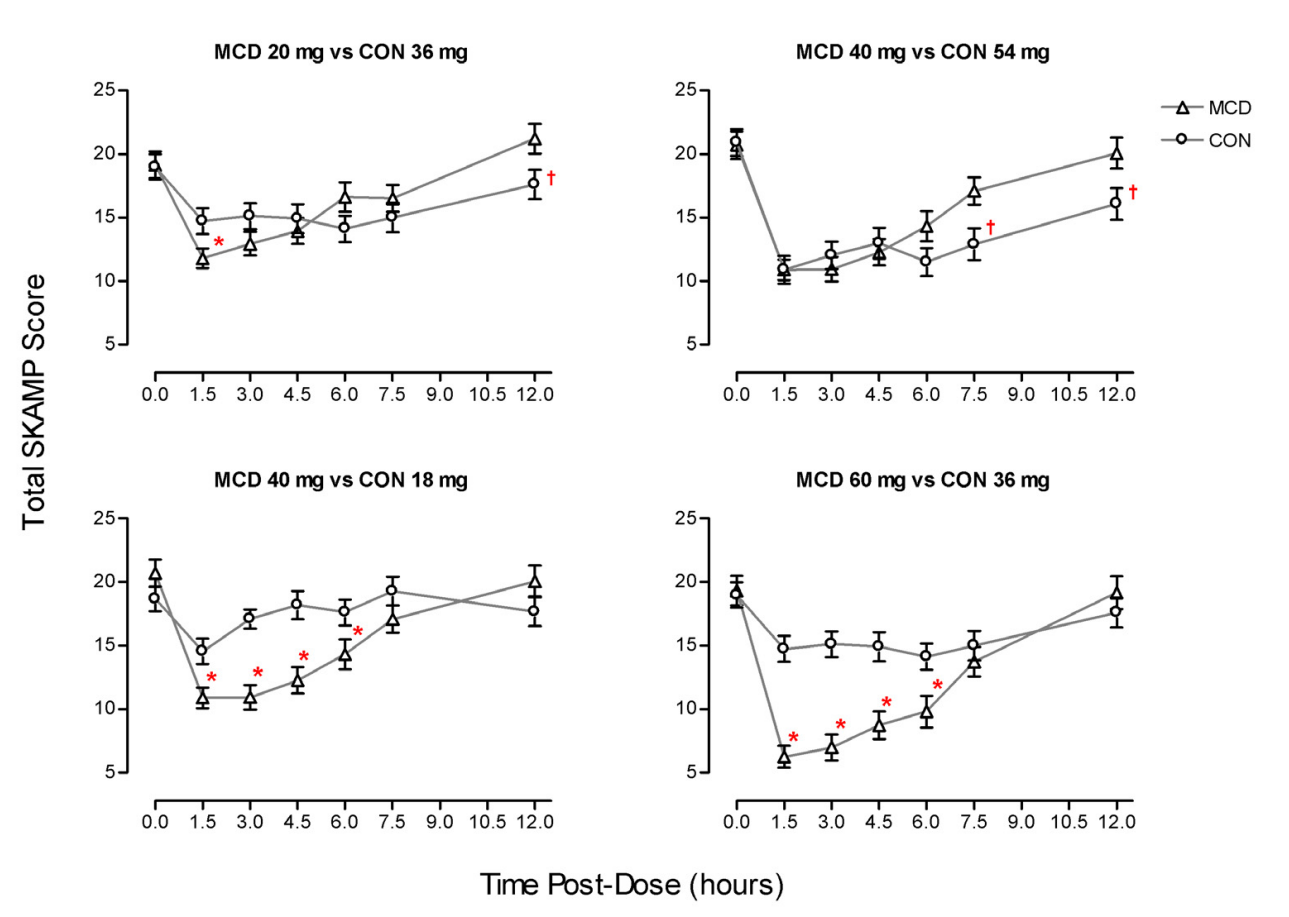
**Mean (± SE) of placebo-adjusted total SKAMP scores for each comparison**. Lower SKAMP scores indicate greater efficacy. Asterisks indicate MCD was significantly better than CON (p < 0.05) while crosses indicate CON was significantly better than MCD (p < 0.05).

The PLA adjusted scores for the total SKAMP score relating to the specific cross-dose comparisons are presented in Figures 1a through 1d. The number of patients included in specific analysis were as follows: MCD 20 vs. CON 36 – 58 vs. 53; MCD 40 vs. CON 54 – 55 vs. 47; MCD 40 vs. CON 18 – 55 vs. 57; and MCD 60 vs. CON 36 – 49 vs. 53.

### Lower daily doses of MCD than CON

The comparison of MCD 20 mg and CON 36 mg revealed no overall difference between treatments, F (1,108) = 0.001; ns. There was an effect of session, F (6,648) = 15.08; p < 0.001 and an interaction between treatment and session F (6,648) = 3.98; p < 0.01. The two treatments did not differ at the time of dose administration or 3, 4.5, 6 and 7.5 hours after it (Fs < 2.5). MCD was superior to CON at 1.5 hours, F (2, 110) = 4.48; p < 0.05, while CON was superior to MCD at 12 hours, F (2,109) = 3.94; p < 0.05. The comparison of MCD 40 mg and CON 54 mg revealed no overall difference between treatments, F (1,99) = 1.05; ns. There was an effect of session F (6,594) = 36.20; p < 0.001 and an interaction between treatment and session, F (6,594) = 3.72; p < 0.01. There was no difference between treatments at the time of administration or at 1.5, 3, 4.5 and 6 hours (Fs < 3.58; ns). However, CON was superior to MCD at 7.5, F (1,100) = 6.26; p < 0.05, and 12 hours, F (1,99) = 4.55; p < 0.05.

### Lower daily dose of CON than MCD

For the comparison of MCD 40 mg and CON 18 mg there was a main effect of treatment with MCD being associated with lower (i.e., better) SKAMP scores than CON, F(1,109) = 6.50, p < 0.05. There was also an effect of session, F(6,654) = 18.61, p < 0.001 and an interaction between treatment and session, F(6,654) = 8.32; p < 0.001. MCD gave lower (better) scores at 1.5 (F(1,111) = 10.02; p < 0.005), 3 (F(1,110) = 18.36; p < 0.001), 4.5 (F(1,111) = 16.16; p < 0.001), and 6 (F(2,111) = 5.94; p < 0.05) hours. However, there was no difference between the two formulations at 7.5 and 12 hours, Fs < 2.75. MCD 60 mg also gave significantly lower scores than CON 36, F (1,99) = 14.03; p < 0.001. Once again there was an effect of session, F (6,594) = 35.38; p < 0.001, and an interaction between treatment and session, F (6,594) = 10.55; p < 0.001. MCD gave significantly lower scores at 1.5 (F (1,100) = 35.01; p < 0.001), 3 (F (1,100) = 30.99; p < 0.001), 4.5 (F (1,99) = 18.23; p < 0.001) and 6 (F (1,100) = 9.66; p < 0.005) hours but not at 7.5 and 12 hours, Fs < 0.55.

## Discussion

CON and MCD are once-daily formulations of MPH using different drug delivery mechanisms resulting in different plasma concentration vs. time profiles in both adults [13] and children [8]. In the COMACS study we compared the PD profiles of equivalent daily doses of the two formulations in children with ADHD [12]. The primary analysis was restricted to a comparison of the relative efficacy of the two formulations within subjects and dosing strata (i.e., across AUC-equivalent total daily doses). As predicted by the PK-PD model proposed by Swanson [19], MCD provided greater symptom control in the morning (from 1.5 through 4.5 hours post-dose), while CON gave greater control in the early evening (at 12 hours post-dose). However, on the basis of the IR/ER ratios of the two formulations, the expected plasma concentration vs. time profiles, and the informal observation of the relative efficacy of the different formulations made between subjects across dosing levels in the COMACS study, it was predicted that a lower total daily dose of MCD (i.e., 20 and 40 mg) would give equal levels of symptom control in the morning when compared to CON at the next highest dosing strata (i.e., 36 and 54 mg); the situation being reversed for CON in the later part of the afternoon and in the evening. Specifically, it was predicted that MCD 20 mg and 40 mg would give similar levels of symptom control to that provided by CON 36 mg and 54 mg, respectively, from 1.5 through 4.5 hours post dose. This would reflect the fifty percent higher relative proportion of IR delivery in MCD compared to CON. In contrast, the higher ER doses of CON would give significantly better control between 6 and 12 hours.

There was only partial support for these predictions. Not only was there no significant difference between placebo adjusted total SKAMP scores for MCD 40 mg and CON 54 mg from 1.5 through 4.5 hours, but also at 6.0 hours post-dose. In addition, comparison at the lower dose levels (MCD 20 mg and CON 36 mg) gave a surprising result: MCD appeared to be associated with a stronger effect and a more rapid onset of action than CON during the very early period post-dose. This was indicated both by the absolute values (i.e., the intercept) for the two formulations at 1.5 hours post-dose and the steeper slopes for MCD than CON between 0 and 1.5 hours. This was a particularly unexpected finding because at the dose levels being compared, MPH available from the IR component of MCD (6 mg) was less than that available for CON (8 mg). Given that the active drug is the same in the two formulations and assuming that clinical efficacy reflects MPH serum concentrations as has been proposed by Swanson [19] this would suggest that non-drug related factors may be involved. Non-drug factors may include those associated with the speed of dissolution and absorption associated with the different delivery mechanisms of the two formulations. For instance, it may be that the IR beads of MCD are associated with a higher rate of dissolution than the CON MPH overcoat, although there is no reason to expect this. Alternatively, the rate of dissolution of the IR components of the two formulations may be equivalent but the distinction between the IR and ER components may be less clear-cut in MCD than CON. This would produce early exposure to MPH from some ER beads in addition to IR beads during the early part of the day. Dissolution studies support this by suggesting that while the ER components of MCD and CON both start to release MPH at approximately 1 hour post-dose, the MCD ER MPH component seems to be more front-end loaded, while CON's ER MPH component seems to be more back-end loaded. MCD drug delivery technology, may therefore, be more efficient at delivering IR components at low doses, and this requires further investigation.

The predictions relating to the efficacy of these different dose comparisons during the latter part of the day were confirmed only at the 12 hour testing session for the lower doses (CON 36 mg vs. MCD 20 mg) and the 7.5 and 12 hours sessions for the higher doses (CON 54 mg vs. MCD 40 mg). Given their expected plasma concentration vs. time profiles in children, one would expect greater efficacy from CON than MCD whatever the dose comparison being made. The 7.5 hour post-dose period is less clear-cut in terms of the relative benefit of the two formulations.

The second set of predictions related to the value of lower daily doses of CON (18 mg and 36 mg) to provide equivalent symptom coverage compared to higher doses of MCD (40 mg and 60 mg, respectively) in the late afternoon and superior coverage in the evening. The predictions for late afternoon were supported at both the higher and lower cross-dose comparisons with lower doses of CON giving equivalent control at 7.5 hours. However, interestingly, at 12 hours the higher doses of MCD remained equivalent to CON. This result is in keeping with the idea, supported by the expected plasma concentration and known dissolution data that MCD continues to release MPH up to 12 hours post-dose. As expected, the higher doses of MCD had a greater effect between dosing through 6 hours post dose.

Given design limitations it is possible that these effects are not 'real' effects related to dose and treatment type but are related to differences between the types of children assigned to different dose levels. The way in which the children were assigned to dose level meant that across-dose comparisons of different treatments could be subject to a number of confounds. First, it could be that children were placed on higher doses because they had a more severe form of ADHD. In the present study we attempted to deal with the possible confounds that such an approach to dose assignment might bring by using the placebo SKAMP score (across all time periods) as a covariate. This score was included as a proxy for severity of ADHD in order to control for the possibility that children assigned to the higher-dose level had a more severe form of the condition.

A second possible confound, not corrected for by controlling for placebo scores, relates to the child's sensitivity to MPH: Children may have been prescribed higher pre-study doses of MPH because they were less sensitive to MPH and did not respond to the lower dose. Such variations in sensitivity could be independent of overall severity of the disorder and therefore constitute a second possible confound. If this were the case then an across-dose comparison would be between more- and less-sensitive children. There are two pieces of evidence that argue against this as an explanation for the current results. First, Swanson et al. [12] reported that the between-subject factor of dose was significant on SKAMP Attention scores in the COMACS study. If dose levels were determined by MPH sensitivity such an effect would not have been expected. Second, there is no reason to believe that sensitivity to MPH should favor one formulation at one time (i.e., MCD in the morning) and the second formulation at another time (i.e., CON in the evening). Differential MPH sensitivity, therefore, seems an unlikely explanation for the current pattern of results. It is also possible that basing the study dose level on pre-study total daily dose may have had differential effects for children receiving TID and BID doses of IR preparations prior to the study. Children on, for instance, two tablets of 10 mg IR given early in the morning and then in the afternoon would be assigned to the low dose strata of 18 mg of CON thought to be equivalent to 5 mg IR tablets taken TID. In this case, children, although receiving AUC-equivalent daily doses, would be receiving a lower morning (5 mg rather 10 mg) and afternoon dose (5 mg rather 10 mg) and a new evening dose (5 mg as opposed to no dose) during the study relative to their pre-study MPH daily treatment. However, in the current study, 91% of children were on once-a-day dosing prior at prescreening, and it is therefore difficult to estimate the effects of this factor on the current across-dose comparison.

Taken together, the existence of these confounding factors and complications means that while these data provide an initial indication of the relative efficacy of different doses of CON and MCD at different points across the day they should be treated with a certain degree of caution, and the results should be confirmed in a study where subjects are randomized to dose level rather than being assigned to it on the basis of their pre-study MPH daily dose and plasma levels of MPH are assessed simultaneously.

## Conclusions

From the point of view of the practicing clinician the results of this study further highlight the value of having available different ER MPH formulations with different expected plasma concentration vs. time profiles offering different patterns of efficacy over different periods of the day. Given the established dose-response relationship between MPH and side effects, clinicians and parents may wish to limit total daily MPH intake as far as possible while maintaining a tolerable level of efficacy over the day as a whole and/or targeting a particularly important period of the day for a particular patient. This head-to-head study makes a first step towards providing the systematic evidence base on which to make such decisions.

## Competing interests

EJB and DC are consultant for and/or have received support from Celltech, McNeil, Jannsen Cilag and Eli Lilly. JMS is a consultant for Celltech, McNeil and Eli Lilly. SJH and HHD are employees of Celltech Americas, Inc.

## Authors' contributions

JMS, DC, SJH and EJB participated in the design of this post-hoc analysis while EJB performed the statistical analysis. HHD and EJB prepared the manuscript. All authors contributed discussion and read and approved the final manuscript.

## Pre-publication history

The pre-publication history for this paper can be accessed here:


